# An analysis of sickness absence in chronically ill patients receiving Complementary and Alternative Medicine: A longterm prospective intermittent study

**DOI:** 10.1186/1471-2458-6-28

**Published:** 2006-02-12

**Authors:** Susanne Moebus, Nils Lehmann, Wolfgang Bödeker, Karl-Heinz Jöckel

**Affiliations:** 1 Institute for Medical Informatics, Biometry and Epidemiology, Medical Faculty, University of Duisburg-Essen, Hufelandstr. 55, 45122 Essen, Germany; 2 BKK Bundesverband – Federal Association of Company Health Insurance Funds, Kronprinzenstr. 6, 45128 Essen, Germany

## Abstract

**Background:**

The popularity of complementary and alternative medicine (CAM) has led to a growing amount of research in this area. All the same little is known about the effects of these special treatments in every-day practice of primary care, delivered by general practitioners within the health insurance system. From 1994 to 2000 more than 20 German Company health insurances initiated the first model project on CAM according to the German social law. Aim of this contribution is to investigate the effectiveness of multi-modal CAM on chronic diseases within primary health care.

**Methods:**

A long-term prospective intermittent study was conducted including 44 CAM practitioners and 1221 self-selected chronically ill patients (64% women) of whom 441 were employed. Main outcome measure is sick-leave, controlled for secular trends and regression-to-the mean and self-perceived health status.

**Results:**

Sick-leave per year of 441 patients at work increased from 22 (SD ± 45.2) to 31 (± 61.0) days within three years prior to intervention, and decreased to 24 (± 55.6) in the second year of treatment, sustaining at this level in the following two years. Detailed statistical analysis show that this development exceeds secular trends and the regression-toward-the-mean effect. Sick-leave reduction was corroborated by data on self-reported improvement of patients' health status.

**Conclusion:**

Results of this longterm observational study show a reduction of sick leave in chronically ill patients after a complex multimodal CAM intervention. However, as this is an uncontrolled observational study efficacy of any specific CAM treatment can not be proven. The results might indicate an general effectiveness of CAM in primary care, worthwhile further investigations. Future studies should identify the most suitable patients for CAM practices, the most appropriate and safe treatments, provide information on the magnitude of the effects to facilitate subsequent definitive randomised controlled studies that will help to position complementary and alternative medicine in health care.

## Background

The dilemma is becoming ever more obvious: although mainstream medicine, science and health policy refuse Complementary and Alternative Medicine (CAM) as being scientific and efficient, the popularity of the use of CAM is at a premium and growing [1-5]. This conflicting situation is, above all, fostered by the still prevailing shortcomings of good scientific evidence for efficacy and effectiveness of CAM procedures, as well as by the increasing chronic diseases within the population – those disease patterns for which mainstream medicine itself acknowledges having no satisfactory solutions.

Against this background, 22 German company health insurance funds (BKK) in the Rhine-Ruhr area initiated a project according to the German social law lasting from 1994 to 2000. Only within this project, health insurances were allowed to pay for CAM therapies.

The scientific evaluation of the project had to be carried out on account of legal requirements. Therefore, an observational study with quality control steps according to standard operation procedures (SOP) has been performed, committing sickness absence as a main study outcome. Sickness absence is widely accepted as an objectively and integrated measure of morbidity in the working population [6-10], though it is still a seldom used outcome measure in epidemiological/clinical studies. Even more seldom are studies which directly link absenteeism as one outcome variable and the individual health status [11].

The purpose of the study is to provide profound information about the effectiveness of CAM in chronically ill patients. The purpose, however, is not to prove that a specific CAM treatment is effective for a specific disease. Rather, it is meant to investigate possible longterm effects in everyday practice in primary care. In this contribution, we examine the overall effectiveness of CAM interventions on trends in sick-leave, together with patients' health related quality of life. Particularly, we investigate the extent of the regression-towards-the-mean effect, an often ignored ubiquitous statistical phenomenon in pre-/post-treatment measurements [12-14].

## Methods

We conducted a longterm prospective cohort study with intraindividual pre/post comparisons using patients' questionnaires, documentations of the participating physicians, and health insurance data.

Study participants were self-selected patients recruited by the involved health insurance funds, the participating physicians and press articles during 1994 – 2000. All individuals gave written informed consent. Due to the assessment of real-life practice in primary care the only inclusion criteria were the membership to one of the participating health insurance funds and the presence of a non-life-threatening chronic disease (e.g. back pain, migraine, skin diseases, allergies) that has not been improved with conventional therapies, or for which, according to medical experience, a cure or relevant improvement could not be expected. All participating CAM practitioners had to be medical doctors and qualified for the CAM treatments they provided. A wide range of CAM procedures for reimbursement have been authorised by the federal insurance office especially for this project (table 1).

Data was collected from different sources: Case report forms for documenting therapy processes and data of routine health insurance records (sickness absence, in hospital, medication). Patients' self-administered questionnaires comprising health status, on the basis of the questionnaire of the German National Health Survey (NHS). The NHS was part of the German Cardiovascular Prevention Study [15], that comprises three nationwide cross sectional surveys in the former West Germany between 1981–1996. The NHS is a major source of the German health reporting system and is provided as public use files, frequently used for scientific research.

Sick-leave days (SLD) and duration of sickness leave per case (DSL) served as indicators for morbidity. The cumulative sick-leave incidence (CSLI, the proportion of patients with any sick-leave in a given observation year) served as ill health indicator for the whole study group. Standardised rate ratios (SRR) for SLD were obtained by dividing sick leave days by the mean values in strata defined by age classes and gender in the reference population. As reference population all members of the German Company Health Insurance Funds were chosen. Sick leave data of the reference population are documented in the annual health reports [16].

**Figure 1:**
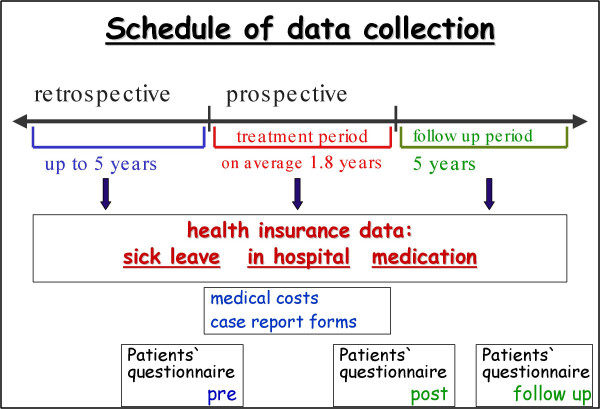
Pattern of data collection.

Patients outcome assessments were done at baseline before start of treatment, at the end of treatment, and at follow up 1.5 years after the end of treatment (figure 1). Health data collection occurred retrospectively up to 5 years, depending on the availability of health records at the health insurance funds for each patient. Time span of prospective data collection depended on duration of therapy (maximum 5 years) and availability of health records in the follow up period (max. 5 years). For purposes of analysis we chose the time span -3 to +4 years, since the years -4 and -5 seemed not to be informative for possible treatment effects. Nevertheless data of the years -4 and -5 were used to calculate regression to the mean effects. For one subgroup analysis we used a narrower range of -2 to +4. Furthermore data of the fifth year of follow up are not presented here, since the number of patients with available data was very small (n = 35) at the time of analysis.

### Statistical analysis

The SLD distribution shows strong positive skewness with 40% of the patients having no sick-leave in a given observation year. Applying a log-transformation reveals an excess of patients with no sick leave in a given observation year. Therefore, we treat the number of patients without sick leave – or its complement, the CSLI per year – as a distinct parameter for sick-leave in the entire study group. The CSLI and measures of SLD and SRR distributions characterise the time dependence of sick leave in the study population. The quartiles of those measures are presented with their 95% confidence intervals (95% CI).

Changes in the CSLI are subjected to a χ^2^-test-of-fit to a uniform distribution over the observation years. Repeated measures multivariate analysis of variance based on ranks was used to explore changes in SLD. The repeated measures design confines analysis to dependent samples, that is, to well defined subcohorts with non-missing sick-leave data in every observation year compared.

The kind of study design – observational long term study including retrospective data, a pre/post comparison, no explicit control group and highly selected patients – suggests an effect of regression-toward-the-mean (RTM) in our data [12,17,18], a statistical phenomenon, first defined by Galton [12]. RTM originates from selection by value and poor correlation of variables, and is of particular importance for repeated measurements as in longitudinal studies. In short: RTM is associated with Pearsons' correlation r between e.g. the measured values of a quantity at one point in time and the values of the same quantity at the next point in time. The effect, observable in subgroups selected by the value of the quantity (for example, the upper 25% of the population at the first point in time) is strongest for zero correlation and vanishes at r = 1. Unawareness of this phenomenon may lead to misinterpretation of data.

In order to check up on RTM we (1) controlled for common societal trends standardising sick-leave data to the population of all German company health insurances (SRR). Assuming that sick-leave five to four years before inclusion to the study is independent from study participation, we (2) estimated the effect of RTM from the fifth to the fourth year in our cohort: the strongly skewed distributed SRR is represented by the classes (SRR = 0, 0<SRR< = 1, SRR>1), correlation is then given by a transition matrix between adjacent points in time (years). We observed that in both remote years five and four years before inclusion, mean SRR in the model experiment was stationary at 0.86 for 321 patients with sick-leave data reported in both these years. The respective 95%CIs are 0.69–1.03 five years and 0.7–1.02 four years before inclusion, indicating comparable sick leave levels of the patients to the level in the reference population five and four years before enrolment. A mean SRR slightly lower unity may be accountable for by demographic characteristics of patients volunteering for CAM treatment. (3) For the present argument, the observed steady state of mean SRR is decisive. We therefore may estimate the matrix describing the process of regression-toward-the-mean in the reference population from the participants' data in the indicated consecutive remote years. Multiplying this estimate for the transition matrix with the vector of class percentages in a given year yields the class percentages in the next year as expected under regression-toward-the-mean. To compare classified SRR distributions between years we employed Cochran-Mantel-Haenszel (CMH) statistics.

Note, that all significance levels associated with the above tests are understood to be strictly descriptive. Analyses were performed with SAS software package.

## Results

Fourtyfour practitioners treated 1221 patients, 441 of whom were employed and might take sick leave. Baseline characteristics are described in table 2. The following results are on the basis of the working cohort. As main diagnosis dorsopathies (23.7%), osteoathrosis/-athritis (9.1%), skin diseases (8.0%), headache/migraine (6.9%), and chronic obstructive lung diseases (5.5%) were documented most often. With the exception of cardiovascular diseases, a similar distribution of diseases can be found in the reference population [17]. Before treatment, the mean duration of disease was 10.6 years, and on average six different conventional therapies with respect to the main diagnosis were recorded.

Standardising our data according to age and sex, study participants represent a higher social status than the NHS population. The greatest differences appeared, as expected, in reference to the health status: patients engaged more in sports, cared more for their health and were to a higher degree convinced that one can influence ones own health status. The current health state was considered to be much poorer, and distress such as backache, pain in neck and shoulders, feeling of weakness, and weariness were more often represented among study participants compared to the German population.

The mean CAM treatment lasted 2.0 years (mean intervention time). Patients were treated on average with three different therapy modalities, with acupuncture, face time, homoeopathy, oxygen therapy, and neuraltherapy as the most often used. Surprisingly, phytotherapy seemed to have only a small meaning, although of the drug groups practitioners prescribed most often, phytotherapeutics ranked on the fourth place with 7%, besides homoeopathics (15%), vitamins/minerals (9%) and bacteria lysates (8%).

Average loss of sick-leave days amounted to 31 (SD ± 61.0) days in our cohort prior to inclusion (table 3) and exceeded absenteeism of the reference population [16]. The distribution of SLD showed a noticeable time dependence in coincidence with CAM treatment. A deep drop of SLD down to 24 days occurred mainly in the second year after enrolment, remaining at this low level in the following years (table 3). Since 80% of treatments lasted less than three years this might indicate a sustainable effect of CAM interventions on sick-leave.

**Figure 2:**
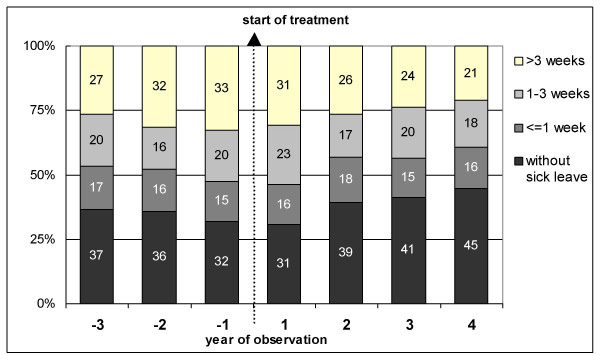
Proportion of patients with given sick leave duration, unstandardised.

**Figure 3:**
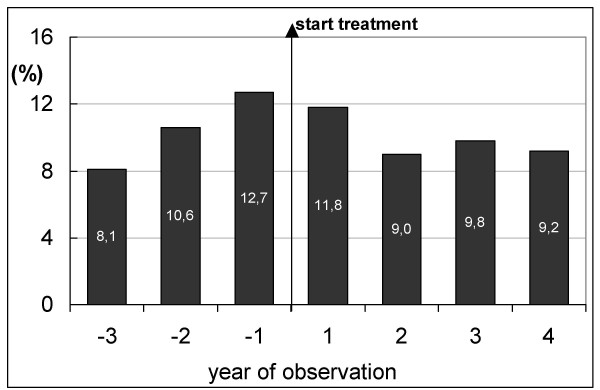
Proportion of patients with longterm sick leave (>six weeks in one given case).

Classifying patients as those without any sick-leave, with durations less than 1 week, 1–3 weeks and longer than 3 weeks in one given observed year, there was a marked shift towards a higher proportion of patients with lower sick-leave durations: 39% in the second year compared to 32% before start of treatment for those with no sick leave and 18% in the second year compared to 15% before start of treatment for those with durations less than 1 week (figure 2). This shift persisted in the following years. Furthermore, the proportion of patients with durations >6 weeks in one given sick-leave case was sustainably lowered (figure 3), which is especially of high economical interest for the health insurances, since by German social law they have to take upon the salary of their members from the 43rd day on.

Table 4 shows the time course of the mean and 75. percentiles (Q_3_) of the SRR in a subcohort of patients with recorded data in all observation years. Taking into account the 95%CIs, mean and Q_3 _were distinctly larger than the reference value SRR = 1 until the first year of treatment. Thereafter in the prospective period, Q_3 _is comparable to unity, which means that 75% of the patients had the same amount or less sick-leave than expected for their age and gender in the reference population.

**Figure 4:**
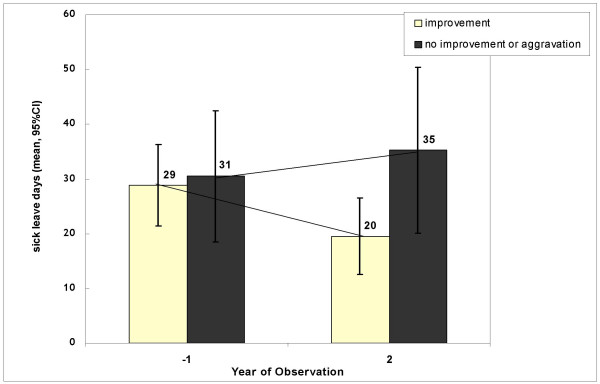
Development of sick leave days (mean and 95% CI) of patients with selfreported improved, unchanged or impaired health status two years after start of treatment.

Stratifying sick-leave data by health improvement as a surrogate indicator for good/poor success of CAM intervention, table 5 shows the proportion of patients with improved versus impaired or unchanged self-reported health status. More than 70% reported an improvement. With regard to sick-leave, figure 4 unmasks a quite revealing pattern of sick-leave development. Before CAM intervention, the SLD distribution is similar in both groups; but in the second year of intervention, the SLD of the improved patients dropped clearly, contrary to non-improved patients.

**Figure 5:**
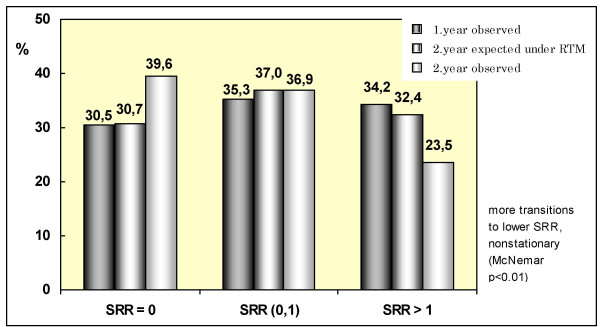
Controlling for regression to the mean. Development of proportions of patients (subcohort, n = 187) with no (SRR = 0), equal (SRR 0,1) or more sick leave (SRR>1) in the first and second year after treatment compared to the reference population with observed and expected proportions (under the assumption of regression towards the mean). Test: 1. vs. 2. year p = 0.01 (CMH), expected vs. 2. year: p = 0.02 (CMH).

Up to this point, the observed development of sick-leave could solely be the spurious effect of regression-toward-the-mean (RTM). Figure 5 shows the observed distributions in SRR in the first year of treatment: 30.5% with no sick leave compared to the population of all German company health insurances (SRR = 0), 35.3% with equal sick leave day (SRR 0,1), and 34.2% with more sick leave (SRR>1). Under regression-toward-the-mean we expect 30.7%, 37.0% and 33.3% in the second year. Nonetheless, the observed SRR-distribution in the study population in the second year is 39.6%, 36.9%, 23.5% (figure 5). This suggests a relevant net decrease of standardised sick-leave days.

## Discussion

Characteristics of our study cohort are typical for user of CAM: On average, they are more likely middle aged, female, multimorbid, higher educated, of poorer subjective health, with chronic pain, non-life-threatening health problems and are high utilisers of the health care system [4,19-22]. The latter item is especially interesting since our outcome parameter, absenteeism, is an internationally established indicator for work related morbidity [6], and was also used to study effects of acupuncture in randomised trials [23]. Furthermore absenteeism imposes considerable direct and indirect costs on the employer and health insurances. Accordingly, the revealed sustained reduction of absenteeism, exceeding regression-toward-the-mean effects and societal trends, might indicate at least an indirect economical effect of the multifaceted CAM intervention, particularly with regard to the reduction of long spells. Together with the improvement of the health status this is a plausible argument for a beneficial treatment for this kind of patients. Up to which degree the observed effects might have an overall economical benefit has yet to be proven.

The observed pattern of sick leave with higher rates of absenteeism in men is different to results of many other epidemiological studies reporting consistently higher levels of absenteeism in women [e.g. [7,24-26]]. This could be due to the patient-based cohort group here, with especially highly selected chronically ill men. Since it is known that women prefer CAM therapies, one can assume, that only those male study members entered the study, showing the highest perceived morbidity. Self-perceived well-being on the other hand is in some way associated with taking sick leave [27].

In Germany, almost all insurance companies initiated projects according to the German social law [28,29]. Although none of them included this wide spectrum of CAM therapies – homoeopathy and acupuncture are mostly involved – those examining sick-leave found similar results [29]. Contrasting to our study, they did not control for regression-toward-the-mean and secular trend. However, the evaluation of these projects will give comprehensive insights into the use of CAM in primary care patients. In the period from 1995–2007 the insurance funds in Germany will altogether have spent approximately € 510 million for CAM treatments and their scientific evaluations (€ 12 mio) [30].

Within this project study physicians were allowed to practice a variety of CAM procedures without financial restriction. On a first glance, this might be quite unfavourable for evaluation purposes. Otherwise, for the first time, we are able to achieve insights of general practices of CAM practitioners in primary care.

Concerning study limitations (1) a major bias might derive from a spontaneous regression of disease, but this seems unlikely given the long-term chronic condition of the patients and the very long observation time. (2) Study subjects were not aware of the fact that the principal outcome would be absenteeism, so it is unlikely to assume that participants were less absent from work because of their knowledge about the main outcome. (3) The observed temporal course of sick-leave could rely only on regression-toward-the-mean, especially with this highly selected group of patients. But again, the long retrospective and prospective time span observed made it possible to control for regression-toward-the-mean, demonstrating an effect that exceeds this phenomenon. (4) A potential bias might arise from different sample sizes at different observations years. However, neither a last observation carried forward nor a complete case analysis resulted in major modifications of our results. (5) It must be taken into account that the feature of being unfit to work is influenced by several confounding factors [31,32]. A remarkable influence on the number of SLDs is the severity of the chronic disease, as well as different kinds of working conditions and workload. Absenteeism is a complex process, and although a poor health status is related to higher rates of absenteeism [33], there are also different coping behaviours that influence taking sick-leave [34]. This phenomenon might explain the relatively high proportion of chronically ill patients without any sick-leave in one given year. Nevertheless, the pattern of our sick-leave data, combined with self-reported health status is consistent with constant effects of the CAM intervention on sick-leave in our cohort. (6) At least, possible co-interventions might have influenced the observed changes. During the observation period patients could have received additional conventional treatments. When asking patients for further therapies, we found that more than 60% received additional treatments, but in most instances these referred to treatments of dentists, gynaecologists, ophthalmologists or orthopaedists, seldom to treatments of radiologists, neurologists, ENTs. Less than 5% of the patients reported co-intervention from other general practitioners. So the impact of co-intervention seemed to be low at a first glance. Nevertheless, when analysing the prescription patterns of the study practitioners, we could find some (modern) conventional pharmaceutical therapies, e.g. Aarane, a bronchodilatator for asthma [35]. Further analysis are planned to describe the diverse strategies of CAM practitioners regarding treatment patterns, number of different CAM specialities and conventional therapies used, treatment durations, costs in association with patients satisfaction and health improvement.

Finally, some comments on the relevance of our results have to be made relating to the observational study design. With the special situation of evaluating a project according to the German social law, we had to regard some conditions that differ from clinical studies. First, establishing a reliable control group was not feasible since patients interested in participating were highly in favour of being treated with this variety of CAM interventions. It is known from other studies that patients with a strong preference for a particular treatment will refuse randomisation [36,37]. What is more, it had to be expected that our chronically ill patients would refuse those conventional therapies which they had already experienced without success years before. Second, logistical reasons excluded a random invitation. Third, no comparable population with definitely no access to CAM was available due to data protection reasons and lack of interest of conventional practitioners to cooperate. The absence of a suitable control group could partly be compensated by the long observation time and by the use of routine health data for comparison, but nevertheless limits the generalisability of our conclusions.

Profound observational studies are pragmatic approaches to questions concerning the practices used for particular diseases, the numbers and types of patients who use them, and how well patients respond to treatment [38]. Therefore, the evaluation of this intervention project reveals comprehensive insights of multi-modal CAM use in general practice. The value and meaning of results of well-designed observational studies have been extensively discussed [39-45] and we agree with Rosenbaum [46], who states that results from observation studies strengthen the evidence, but do not prove the treatment caused its ostensible effects. We are well aware of the fact that this study by no way could adress the question of efficacy (efficacy here refers to an intervention that has been shown to be superior to placebo in randomised controlled trials) of any of the CAM treatments applied. It is meant to show possible long-term effects in association with the special intervention in real-life practice. The definition of a relevant endpoint, the confirmation of a study protocol with a clear study question and the provision for quality assessment as prerequisites of a careful observational study [47], make this study an approach in which an investigation of a whole system has been undertaken in its proper context.

## Conclusion

Extensive research in CAM is needed, that will help to define the place of these interventions in health care. This is called for, seeing as the continuing public demand for CAM, combined with still missing sound scientific research, will affect and challenge health care delivery.

From a public health perspective we definitely need to answer questions such as: Which procedures of CAM are safe and effective and for which group of patients? How does CAM meets health care needs, and is CAM associated with high usage of all type of health care? The answering of these questions has to consider social, cultural, political, and economic contexts to maximize the contribution of CAM to the health care system globally. Therefore we need the whole scope of scientific methods available – experimental and randomized controlled trials to outcome studies – in order to be able to make informed and rational decisions within the health care system. What is urgently needed to cope with these challenging tasks is a comprehensive and targeted funding for CAM research, which is almost completely missing in most countries. Altogether this study provides another small building block for the long-term objective of a pragmatic integration of mainstream medicine and CAM that better meets the needs and wishes of all patients – not only in Germany.

## Abbreviations

CAM = complementary and alternative medicine, CI = confidence interval, CSLI = cumulative sick-leave incidence, DSL = duration of sickness leave per case, ENT = ear nose and throat, NHS = German National Health Survey, RTM = regression-toward-the-mean, SLD = sick-leave days, SRR = standardised rate ratio

## Competing interests

The author(s) declare that they have no competing interests.

## Authors' contributions

SM carried out the study and contributed to the acquisition of data, interpretation of data, and drafted the manuscript. NL performed the statistical analysis. WB has been involved in the interpretation of data, and statistical analysis. KHJ conceived of the study, and participated in its design and helped to draft the manuscript. All authors read and approved the final manuscript.

## Pre-publication history

The pre-publication history for this paper can be accessed here:



## Figures and Tables

**Table 1:** Specifically approved Complementary and Alternative Medicine Procedures*

**Diagnostic Procedures**	**Therapeutic Procedures**
holistic anamnese (up to 1 hour)	acupuncture
decoder	electric acupuncture (except: according to Voll)
„Lüscher" test	neuraltherapy
regulation thermography	homoeopathy
electric acupuncture (not by Voll)	colon therapy
	oxygen therapies except: oxyon-, hyperbaric oxygen- and oxygen multistep-therapy
	manual therapy
	reflexzone therapy
	orthomolecular medicine
	isotherapy
	symbiosis regulation

* Especially for this project approved procedures, most of them outside of the regular health insurance reimbursement system. Further procedures like phytotherapy, face time were regular procedures regarding reimbursement at that timenot needing special approval.

**Table 2:** Baseline characteristics of the study population

	**Whole study population (n = 1221)**	**Study population at work (n = 441)**
Women (%)	63.9	54.9
Age (years), mean (± SD)	42.5 (17.4)	41.8 (10.5)
Duration of disease (years), mean (± SD)	10.1 (9.7)	10.6 (9.9)
Duration of treatment (years), mean (± SD)	1.9 (1.2)	2.0 (1.2)
Health status*, mean (± SD)		
Before treatment	3.2 (0.86) (response: 93.9 %)	3.2 (0.82) (response: 97.5 %)
After treatment	2.7 (0.91) (response: 74.3 %)	2.6 (0.91) (response: 74.6%)
Follow up**	2.6 (0.94) (response: 51.1 %)	2.6 (0.88) (response: 54.7%)

* score built from following items: frequency and duration of pain, state of health, degree of impairment, range from 1 – 5, from 1 = excellent to 5= poor health.

** 1.5 years after treatment.

**Table 3:** Sick leave days per patient and year, stratified by sex (unstandardized)

**Year**	**Sick leave days/patient/year**								
	**All**			**Women**			**Men**		

	N*	Mean (SD)	Median (95%CI)	n	Mean (SD)	Median (95%CI)	n	Mean (SD)	Median (95%CI)

-3	369	22 (45.2)	5 (4; 9)	193	19 (40.7)	6 (4; 9)	176	24 (49.8)	5 (3; 10)
-2	404	25 (47.5)	7 (4; 9)	216	22 (33.0)	8 (5; 12)	188	29 (59.9)	4 (1; 9)
-1	441	31 (61.0)	9 (6; 12)	242	29 (58.2)	8 (5; 12)	199	33 (64.2)	10 (6; 14)
1	441	28 (57.5)	9 (7; 11)	242	26 (56.8)	8 (5; 11)	199	30 (58.4)	11 (7; 16)
2	397	24 (55.6)	5 (3; 7)	218	24 (51.3)	5 (3; 8)	179	25 (60.5)	5 (0; 8)
3	297	21 (47.0)	5 (2; 7)	159	18 (34.9)	5 (1; 8)	138	25 (57.8)	5 (0; 10)
4	196	20 (46.1)	4 (0; 6)	102	21 (12.9)	5 (0; 9)	94	20 (50.5)	1 (0; 5)

*Note: Since participants entered the study at different points of time, the follow up period could not be equal for all patients, which causes a drop of observed participants especially in the third and fourth year.

**Table 4:** Standardized Rate Ratio (SRR) of the sick leave quota*

**Observation year^1^**	**Standardized Rate Ratio**				
	Mean	95%CI^2^	Q3^3^	95%CI^2^	P^4^

-2	1.19	0.82;1.57	1.33	0.95;1.78	0.26
-1	1.44	1.05;1.82	1.52	1.18;1.88	-
1	1.45	0.92;1.98	1.34	1.10;1.64	0.90
2	1.04	0.67;1.42	0.96	0.65;1.36	0.03
3	1.12	0.69;1.54	1.01	0.76;1.48	0.01
4	1.10	0.71;1.54	1.01	0.75;1.52	0.02

* Subcohort of patients at work with nonmissing data (n = 187), standardised to German company health insurance population, stratified by calendar year, sex and age.

^1 ^time period with regard to the time of beginning of the treatment of the patient (-3 = observation year three years before, 1 = first observation year after treatment and so one);

^2 ^95% confidence intervall;

^3 ^75. Percentile, a SSR-Q3 of 1.0 means that in that given observation year 25% of the observed patients show the same sick leave durations as in their corresponding reference group regarding age and gender;

^4 ^P-value contrasted to the observation year -1.

**Table 5:** Proportion of patients with improved versus unchanged or impaired selfreported health status after treatment

**Health status after treatment**	**All**		**Men**		**Women**	
	**n**	**%**	**n**	**%**	**n**	**%**
improved	229	71.1	92	66.7	137	74.5
Unchanged/impaired	93	28.9	46	33.3	47	25.5
All	322	100	138	100	184	100

Score built from items '"frequency of pain" and "duration of pain", "state of health", degree of "impairment".
